# Quantitative susceptibility mapping (QSM) of the cardiovascular system: challenges and perspectives

**DOI:** 10.1186/s12968-022-00883-z

**Published:** 2022-08-18

**Authors:** Alberto Aimo, Li Huang, Andrew Tyler, Andrea Barison, Nicola Martini, Luigi F. Saccaro, Sébastien Roujol, Pier-Giorgio Masci

**Affiliations:** 1 Scuola Superiore Sant’Anna, Pisa, Italy; 2 Fondazione Toscana Gabriele Monasterio, Pisa, Italy; 3 School of Biomedical Engineering and Imaging Sciences, King’s College London, London, UK; 4 Department of Biomedical Engineering, School of Imaging Sciences & Biomedical Engineering, King’s College London, St Thomas’ Hospital, 4th Floor Lambeth Wing, London, SE1 7EH UK

**Keywords:** QSM, Cardiac magnetic resonance, Magnetic susceptibility, Heart, Cardiovascular disease, Iron

## Abstract

Quantitative susceptibility mapping (QSM) is a powerful, non-invasive, magnetic resonance imaging (MRI) technique that relies on measurement of magnetic susceptibility. So far, QSM has been employed mostly to study neurological disorders characterized by iron accumulation, such as Parkinson’s and Alzheimer’s diseases. Nonetheless, QSM allows mapping key indicators of cardiac disease such as blood oxygenation and myocardial iron content. For this reason, the application of QSM offers an unprecedented opportunity to gain a better understanding of the pathophysiological changes associated with cardiovascular disease and to monitor their evolution and response to treatment. Recent studies on cardiovascular QSM have shown the feasibility of a non-invasive assessment of blood oxygenation, myocardial iron content and myocardial fibre orientation, as well as carotid plaque composition. Significant technical challenges remain, the most evident of which are related to cardiac and respiratory motion, blood flow, chemical shift effects and susceptibility artefacts. Significant work is ongoing to overcome these challenges and integrate the QSM technique into clinical practice in the cardiovascular field.

Spatial variations in magnetic susceptibility distort magnetic fields. These magnetic field distortions commonly lead to undesirable artefacts in magnetic resonance imaging (MRI) images, but since magnetic susceptibility is related to the underlying composition of tissue it can also provide a wealth of information. One clinically efficient approach to quantifying magnetic susceptibility is to first calculate maps of the main (B_0_) magnetic field, which has been distorted by the varying magnetic susceptibility of the tissue. The magnetic susceptibility distribution is then calculated from the field map, by solving a ‘field-to-source’ problem. Calculation of quantitative susceptibility maps (QSM) cannot be achieved with magnitude gradient-echo images alone, instead phase images acquired at different echo times are needed to measure the B_0_ maps, by first calculating the rate of phase accrual at each voxel (which is equivalent to the local frequency), then converting this to local B_0_ using the Larmor equation. Although this method is technically challenging, owing to the difficulty of obtaining high fidelity B_0_ maps and successfully performing the field-to-source reconstruction, QSM can provide key information on the biochemical constituents and microstructure of tissue. So far, QSM research has primarily focused on applications in neurology, particularly the identification of iron containing substances such as hemosiderin associated with hemorrhage, iron more generally, and paramagnetic contrast agents [1–7]. However, more recently, major advances have also been achieved in QSM outside the central nervous system, including the abdomen [8–10], neck [11] and cardiovascular system. This review provides a critical overview of cardiovascular QSM, as a complementary technique to state-of-the-art multi-parametric cardiovascular magnetic resonance (CMR), exploring how it can improve disease phenotyping and pathophysiological understanding (Fig. 1).

**Fig. 1:**
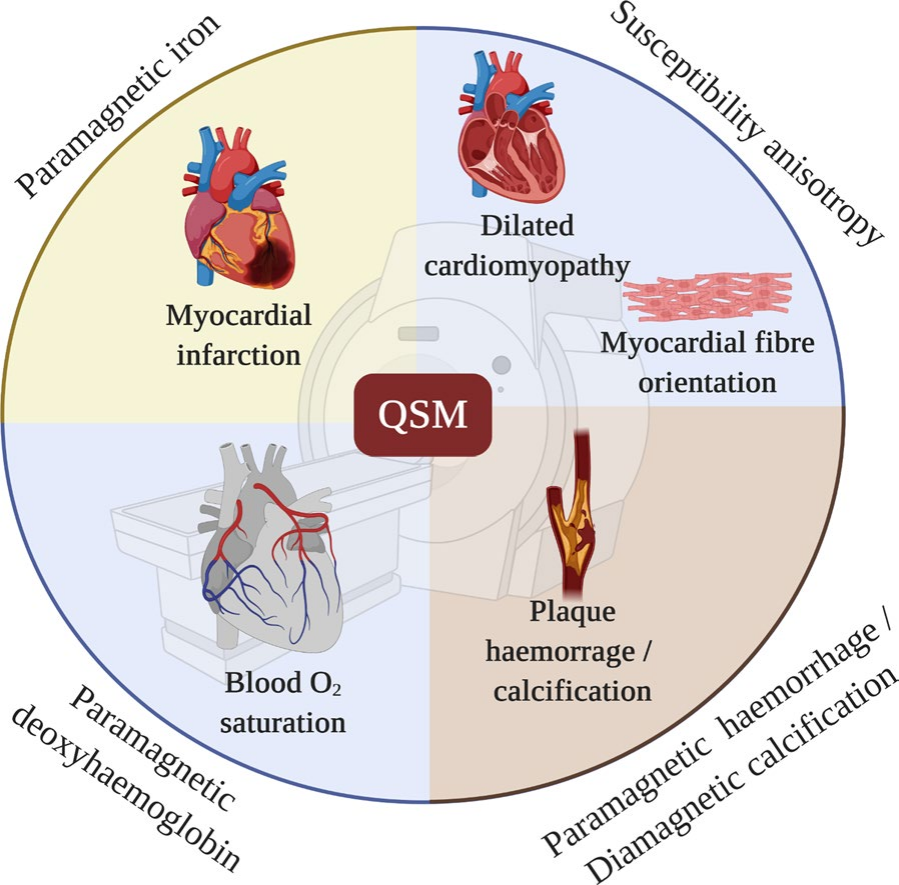
Current applications of quantitative susceptibility mapping (QSM) to the study of cardiovascular disease

## Basic principles

Magnetic susceptibility measures the degree to which a material is magnetized when exposed to a homogenous external magnetic field (H), and is expressed as a dimensionless ratio (χ) between material magnetization (M) and the external magnetic field strength (H), i.e., χ = M/H [12]. Magnetic materials are classified as either diamagnetic (χ < 0), paramagnetic (0 < χ < 0.01), or ferromagnetic (χ > 0.01) [13]. Magnetic susceptibilities are often expressed in parts-per-million (ppm), relative to the magnetic susceptibility of water (which has an absolute magnetic susceptibility of 9 × 10^–6^), this convention will be used for the remainder of the article. Biological tissues can be diamagnetic (e.g., calcium phosphate), paramagnetic (e.g., deoxyhemoglobin, copper or manganese) or ferromagnetic (iron). In CMR, the χ of an organ within the static magnetic field B_0_ can be quantified by mapping the magnetic field perturbation (δB). To map χ, we first construct the forward problem that expresses how the inhomogeneous susceptibility (χ) distorts the magnetic field B_0_, after which the inverse problem is solved. The magnetic field distortion δB(***r***) induced by a susceptibility distribution χ(***r***) can be computed by convolving the susceptibility distribution by the unit magnetic dipole kernel d(***r***), resulting in the following equation [14]

1 $$\delta B\left({\varvec{r}}\right)={B}_{0}\cdot d\left({\varvec{r}}\right)\otimes \chi \left({\varvec{r}}\right),$$

where B_0_ is assumed to be along *z*-direction, ⊗ is the convolution operator, and $d\left({\varvec{r}}\right)$ is defined as

2 $$d\left({\varvec{r}}\right)=\frac{1}{4\pi }\cdot \frac{3{cos}^{2}\theta -1}{{\left|{\varvec{r}}\right|}^{3}}$$

with |***r***| and θ being the radial distance and polar angle in the spherical polar coordinate system, respectively. The induced magnetic field perturbation δB map can be calculated from a Larmor resonance frequency shift (δω) map (measured using 3D multi-echo GRE phase images) with the following relation

3 $$\delta B({\varvec{r}})=\delta \omega ({\varvec{r}})/\gamma ,$$

where γ is the gyromagnetic ratio.

Due to the spatial extent of d(**r**), the measured δB depends on the magnetic susceptibility both within, and outside of the object, making it very challenging to solve Eq. 1. δB is therefore separated into two components, referring to the B_0_ perturbations caused by magnetic susceptibilities located within (δB_loc_), and outside (δB_bkg_) of the object with

4 $$\delta B={\delta B}_{loc}+{\delta B}_{bkg}.$$

Since ${\delta B}_{loc}$ results from the magnetic susceptibility contribution inside the region of interest (ROI), the tissue magnetic susceptibility (${\chi }_{tis}$) inside the ROI can be calculated with the equation

5 $${\delta B}_{loc}\left({\varvec{r}}\right)={B}_{0}\cdot d\left({\varvec{r}}\right)\otimes {\chi }_{tis}\left({\varvec{r}}\right),$$

which can be expressed under the k space formalism as [15].

6 $${\delta B}_{loc}\left({\varvec{k}}\right)={B}_{0}\cdot d\left({\varvec{k}}\right)\cdot {\chi }_{tis}\left({\varvec{k}}\right),$$

by using the Fourier convolution theorem ($FT\left(a\otimes b\right)=FT(a)\cdot FT(b)$) [16], where

7 $$d\left({\varvec{k}}\right)=\frac{1}{3}-{\left(\frac{{k}_{z}}{k}\right)}^{2}$$

is the dipole kernel. The 3D ${\chi }_{tis}$ map is reconstructed by solving this ‘field-to-source’ inverse problem. Unfortunately however, the presence of two conical surfaces at polar angles of ± 54.7° to the z-axis where $d\left({\varvec{k}}\right)=0$, prevents direct deconvolution of Eq. 6 to find ${\chi }_{tis}$ and necessitates the use of either the Calculation Of Susceptibility through a Multiple-Orientation Sampling (COSMOS) method [17] (which is impractical due to the requirement to acquire data with the patient in multiple positions) or regularization [18–26] when solving Eq. 6.

**Fig. 2:**
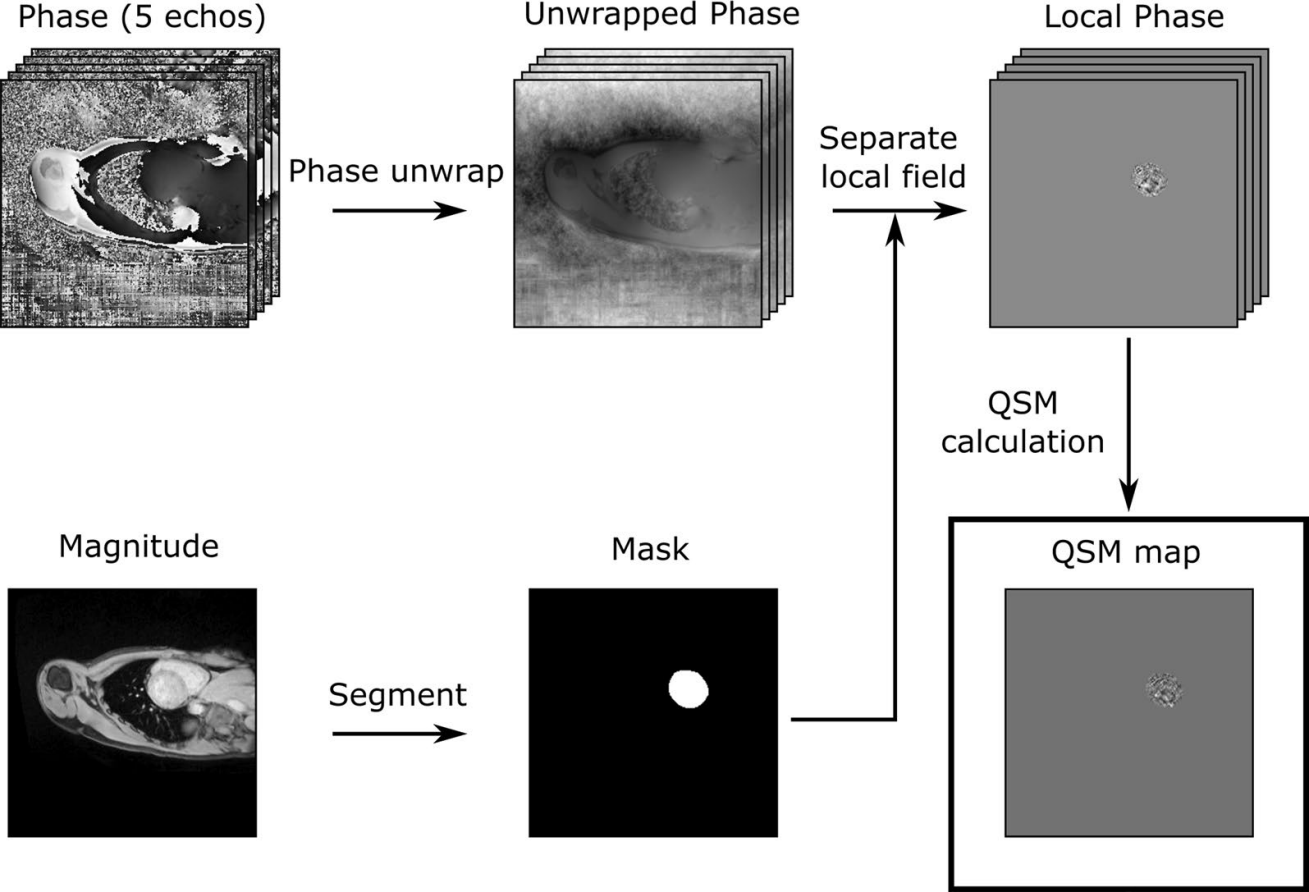
Steps of quantitative susceptibility mapping (QSM) post-processing. Example left ventricular QSM maps were computed from magnitude and phase multi-echo gradient echo images. Step 1: Phase images are phase unwrapped. Step 2: The local and background fields are separated, using segmentation masks generated from the magnitude images. Step 3: The QSM map is computed from the local magnetic field map

In practice, for cardiac QSM, there are three steps to finding this 3D ${\chi }_{tis}$ map which are shown in Fig. 2.

1. Step 1: Estimation of the magnetic field perturbation map. A 3D $\delta \omega$ map is the first estimated from the phase evolution in 3D multi-echo gradient echo (GRE) CMR images; this 3D $\delta \omega$ map is then used to generate a 3D δB map according to Eq. 3. Phase unwrapping is then performed, to remove phase discontinuities, using a variety of methods including, region growing [27, 28], Laplacian [29, 30], and Best-path [31, 32] methods.
2. Step 2: Estimation of the local magnetic field perturbation map. A 3D δB_loc_ map is computed by removal of δB_bkg_ from the δB map based on Eq. 4, using methods such as Sophisticated Harmonic Artifact Reduction for Phase (SHARP) [33, 34], Projection onto Dipole Fields (PDF) [35], or the Laplacian Boundary Value (LBV) method [36].
3. Step 3: Estimation of the tissue magnetic susceptibility map. A 3D ${\chi }_{tis}$ map is reconstructed from the 3D δB_loc_ map by solving the field-to-source inverse problem based on Eq. 6. Using ℓ_1_- and ℓ_2_-regularization techniques such as Morphology Enabled Dipole Inversion (MEDI) [25] or Homogeneity-Enabled Incremental Dipole Inversion (HEIDI) [26].

## Biochemical foundations of QSM

QSM measures the spatial distribution of χ produced by the diamagnetic, paramagnetic and ferromagnetic constituents of tissue [6]. The body contains trace amounts of several paramagnetic transition metal ions such as copper, manganese, and cobalt. Iron is ~ 30 times more abundant in the body than all the other paramagnetic ions together, with an iron content of ~ 53 mg/kg in healthy subjects. As such, a 70 kg human contains ~ 3700 mg of iron, of which nearly 2500 mg are linked to haemoglobin in blood or myoglobin in skeletal muscles (heme iron), with the remainder linked to ferritin or hemosiderin (non-heme iron) [37, 38]. The magnetic susceptibility perturbations seen in human tissue depend almost entirely on non-heme iron concentration [38]. However, in healthy subjects, non-heme iron deposits are evenly distributed throughout the body and therefore result in negligible susceptibility variations. Only when local iron deposits equal or exceed ~ 1 mM in concentration, will a significant χ difference, compared to iron-free regions be observed with QSM, for instance of ~ 1 ppm in severe transmural myocardial infarction [39].

Although T_2_* magnitude imaging by multi-echo GRE is already a well-validated technique to quantify iron deposits, T_2_* relaxation time (or R_2_* = 1/T_2_*) is influenced by local background susceptibility and other sources alongside iron deposits [40]. For instance, in brain imaging T_2_* (or R_2_*) relaxivity, measured by GRE images, can miss pathological iron accumulation due to the competing effect of the diamagnetic myelin, the main white matter component [41]. The magnetic susceptibility of tissue depends upon its constituents, with each constituent’s contribution depending on its electronic structure and concentration. On a molecular level, magnetic susceptibility is determined by the electronic configuration, with unpaired electrons contributing to paramagnetism. However in bio-molecules, this relationship may be less intuitive, for instance, oxyhaemoglobin, which is made up of four globular proteins each containing the Fe^3+^-haeme, is diamagnetic despite Fe^3+^, in aqueous solution, possessing five unpaired electrons, whereas deoxyhaemoglobin is paramagnetic [42].

## Cardiovascular applications of QSM

The application of QSM for studying the cardiovascular system offers an unprecedented opportunity to gain a better understanding of the pathophysiological changes associated with cardiovascular disease. A number of state-of-the-art applications are described in the following sections, each of which has the potential to significantly improve patient care and outcomes in impactful areas of cardiac medicine. Despite this, the field of cardiac QSM is in its infancy, so a number of challenges remain to be solved. The most evident of these challenges are related to cardiac and respiratory motion, blood flow, chemical shift effects at the boundary between the epicardium and epicardial fat, and susceptibility artefacts at the myocardium-lung interface [39, 43].

### Blood oxygenation

Oxygen saturation in the arterial blood (SaO_2_) is a relevant biomarker in many cardiovascular diseases, and is commonly used for the identification and quantification intra-cardiac shunts in congenital heart disease. This metric also provides an index of systemic oxygen delivery and consumption in heart failure [44–46] and pulmonary hypertension [47, 48]. Recently, Wen et al. demonstrated the possibility to gauge venous SaO_2_ by measuring the difference in magnetic susceptibility between the venous and arterial blood pools using QSM [43]. For this purpose, the authors used a multiple breath-hold electgrocardiogram (ECG)-gated 2D multi-echo GRE to obtain T_2_* source images that were then post-processed to generate QSM maps. The latter showed strong differential susceptibility (Δχ) between RV and LV blood pools as a result of the paramagnetic and diamagnetic properties of the deoxygenated and oxygenated hemoglobin, respectively, which allowed them to measure of the blood oxygenation difference (ΔSaO2) using an established formula [43]:

$$\Delta SaO2=\frac{\Delta \chi }{4H\chi deoxyheme}$$

where *4χ*_*deoxyheme*_ is the molar susceptibility of a fully deoxygenated deoxyhemoglobin (151.054 ppb ml/µ) and *H* is the heme concentration in blood, which is derived from the patient’s measured hematocrit (*Hct*), the mass concentration of hemoglobin in a red cell (*ρ*_*RBC;Hb*_ = 0:34 g/ml), and the molar mass of deoxyhemogloblin (*M*_*Hb*_ = 6444·10^–6^ g/µmol) [49].

**Fig. 3:**
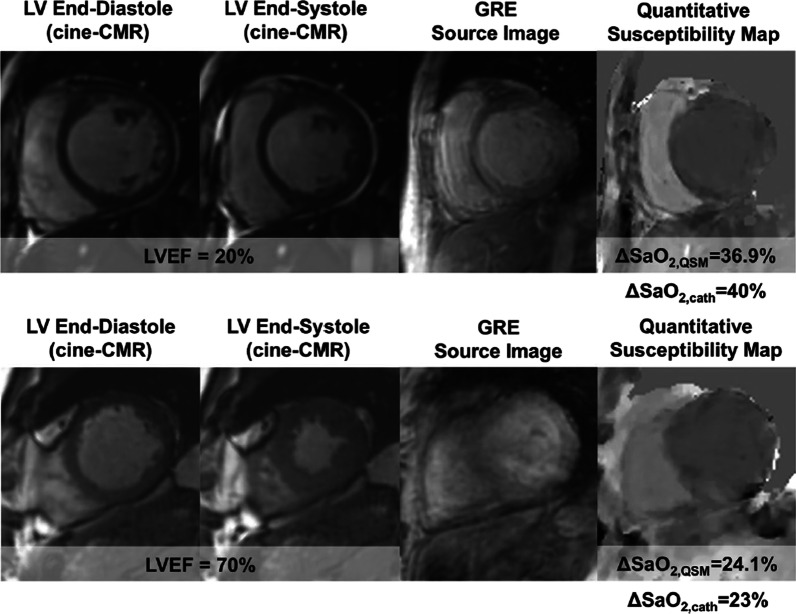
Free breathing three-dimensional cardiac quantitative susceptibility mapping (QSM) in cardiac patients. Representative examples of QSM maps in two cardiac patients which are used to quantify ventricular blood oxygenation. Top: QSM map of patient heart with reduced left ventricular ejection fraction (LVEF) shows a marked difference in blood oxygen between left and right ventricles. Bottom: QSM map of patient heart with normal LVEF shows a difference in blood oxygenation between the ventricles within the normal range (Reprinted with permission from Wen et al. [50])

This approach, however, was complicated by long acquisition time, low signal-to-noise ratio (SNR) and inconsistent positioning of consecutive short-axis slices across the ventricles which lead to non-interpretable source GRE images in 36% of subjects [43]. These limitations were resolved by adopting a free-breathing ECG-triggered navigator-gated 3D multi-echo GRE sequence which allowed the authors to generate interpretable QSM maps in all healthy subjects and 87% of patients with a reduction of acquisition time by about 30% as compared to the 2D approach (Fig. 3). In a clinical cohort (n = 34), patients with left ventricular (LV) dysfunction (LV ejection fraction < 50%) had greater ΔSaO_2_ than those with preserved systolic function. Remarkably, the 3D approach also showed an excellent correlation) and small bias when compared to invasively measured ΔSaO_2_ (gold-standard) in 15 patients undergoing cardiac catherization [50]. QSM-oximetry may offer advantages to conventional methods, which rely on the T_2_, T_2_*, and T_1_ relaxation times of the blood pool. For instance, the dependence of T_2_ relaxation time on blood oxygenation is well established, but requires measuring multiple complex Luz–Meiboom model parameters, restricting its clinical applicability [51]. Recently, Varghese et al. introduced a promising technique that used multiple T_2_ measurements, with different inter-echo pulse spacing, to estimate all the parameters in the Luz-Meiboom model and non-invasively estimate the SaO_2_ [51, 52]. However, this method has shown flow dependency, with signal loss in the specific regions of the cardiac chambers, which combined with the 2D single-slice approach, leads to a dependency on user input during the acquisition and post-processing stages, with ensuing high intra- and inter-observer variability.

QSM-oxymetry has the potential to play a key role in congenital heart disease, where CMR is increasingly used for pre- and post-operative management, as an alternative to invasive catheterization. The non-invasive, ionizing-radiation free, nature of QSM-oximetry is well suited for repeated CMR exams of young patients, complementing phase-contrast velocity-encoding techniques for intra-cardiac shunt diagnosis, management, and follow-up. In addition, QSM-oximetry may provide key information in patients with heart failure or pulmonary hypertension, as well as in establishing the cause of hypoxemia, and may potentially be used to tailor therapy and monitor treatment. Mixed venous oxygen saturation (vSaO_2_) reflects the balance between oxygen delivery and consumption; in patients with heart failure a low vSaO_2_ is associated with advanced and decompensated hemodynamic status due to insufficient cardiac output, and/or reduced arterial oxygen saturation (aSaO_2_). It is important to acknowledge that a substantial number of heart failure patients can have a reduced vSaO_2_ despite a normal cardiac output [53]. Importantly, unlike cardiac output, sSaO_2_ has been shown to predict clinical outcomes in patients with acute myocardial infarction and patients with primary pulmonary hypertension [54, 55].

### Myocardial iron

In patients with ST-segment elevation myocardial infarction (STEMI), re-opening of the infarct-related artery is a prerequisite to salvaging the ischemic myocardium. However, reperfusion per se brings about myocardial damage (ischaemia/reperfusion injury) partially negating the benefits of infarct-related artery re-opening. Intramyocardial haemorrhage (IMH) is a hallmark of severe ischemia–reperfusion injury, occurring in about half of patients with a STEMI. This phenomenon is due to the loss of coronary microvasculature integrity with ensuing erythrocyte extravasation and iron accumulation in the myocardium from hemoglobin breakdown. Compelling evidence indicates that IMH is a strong independent predictor of adverse LV remodelling and untoward clinical outcomes in STEMI patients, potentially due to the increased production of reactive oxygen species promoted by elevated iron levels [56, 57]. This makes IMH, which can be measured indirectly via iron concentration with QSM, an ideal biomarker for testing novel cardioprotective strategies to mitigate ischemia–reperfusion injury. In a swine model of haemorrhagic infarction, QSM showed a paramagnetic shift in the infarct, reflecting an elevated tissue iron content, which was independently validated by histology, plasma optical emission spectrometry, electron paramagnetic resonance spectroscopy, and RNA analysis of markers for iron metabolism. When compared with standard iron-sensitive sequences, including T_2_*-weighted, T_2_*- or R_2_* maps, QSM showed higher diagnostic accuracy for pathological iron overload. For instance, in animals with permanent coronary occlusion QSM showed a paramagnetic shift in the infarct region, compared to a remote myocardium region, where abnormal iron concentration was confirmed by histology, spectrometry, and spectroscopy in the infarct region. This information was missed by standard T_2_*-weighted and T_2_*- or R_2_* (1/T_2_*) maps, which are currently used to detect and quantify IMH. This finding is not unexpected since multiple biochemical factors can exert opposite effects on T_2_* relaxation time in the acutely infarcted myocardium, including shortening due to iron, and lengthening due to edema, fat and collagen deposition. QSM however, directly probes the local magnetic susceptibility [58], which closely relates to iron concentration. This experimental proof-of-concept was replicated in a small cohort of STEMI patients, who underwent a comprehensive CMR 3 days (on average) after the re-opening of the infarct-related artery. In this cohort, the magnetic susceptibility of the infarct was significantly higher than the susceptibility of the remote myocardium for all patients [39], an important finding given the clinical relevance of IMH.

QSM may also be applicable to the assessment of patients with hemochromatosis in order to quantify myocardial iron deposition, which strongly affects LV remodelling and clinical outcome [59, 60]. Both T_1_ and T_2_* maps have been used for myocardial iron quantification, although T_1_-mapping is likely better placed to capture the very early stage of cardiac hemosiderosis [61]. QSM holds the potential of representing an alternative for iron quantification in the heart, but will require validation before it can be used in the clinic, particularly given the dual-challenges of cardiac and respiratory motion. Nonetheless, seminal papers have shown that QSM is accurate, and well-suited to quantifying iron concentration in the liver, with the inherent advantage of measuring the iron concentration based upon a fundamental property of the tissue, i.e., local magnetic susceptibility, rather than extrapolating this information from empirical relaxation parameters (e.g., R2 and R2*), which are affected by other factors including fibrosis and steatosis [62]. QSM reconstruction can account for the different spectral peaks of adipose tissue to ensure an accurate estimation of the B_0_ field maps which are minimally influenced by liver fibrosis or inflammation. Fat fraction and chemical shift corrected magnetic susceptibility maps can be calcutated with methods such as Chemical QSM, which uses the Dixon-based IDEAL method [63]. Moreover, QSM removes the blooming artefacts in regions near the air-tissue or fat-tissue interface that typically affect R2* maps. Collectively, QSM appears to have comparative advantages to the current R2* maps used for liver iron quantification [62, 64] although further validation is required. Finally, it is also important to bear in mind that R2* and QSM maps are not mutually exclusive techniques since either map is reconstructed from source images acquired by 3D multi-echo GRE sequence.

### Myocardial fibre orientation

The restrained distribution of molecules in a specific tissue can be gauged by magnetic susceptibility anisotropy using susceptibility tensor imaging (STI), which calculates a susceptibility tensor using multiple images with different B_0_ orientations. Susceptibility anisotropy originates from the ordered (restrained) spatial arrangement of molecules with diamagnetic (e.g., calcium) and paramagnetic (e.g., iron) properties within a tissue, which then generates a bulk (macroscopic) anisotropic susceptibility. This is exemplified by the ordered distribution of phospholipids in the myelin sheaths which encapsulate axons in the central nervous system, giving rise to measurable macroscopic susceptibility anisotropy [65].

Cardiac myofibrils consist of serially repeated sarcomere units, a highly organised structure of repeated of thick and thin myofilaments oriented parallel to the long axis of the myofibril. This myocardial microstructure is altered in cardiomyopathy, myocardial infarction, and congenital heart diseases, where the initial misalignment of myocardial fibres causes increased myocardial stress, which begets further fibre misalignment and promotes adverse cardiac remodelling, eventually culminating in heart failure or life-threatening ventricular arrhythmias [66–70]. Hence, mapping myofibre organization could be an important tool for assessing the functional properties of healthy and diseased hearts. Ex-vivo, STI is able to capture myofibre orientation by harnessing the magnetic anisotropy of polypeptide bonds in the myofilaments which collectively generate bulk (macroscopic) anisotropic susceptibility on a scale measurable by QSM [71]. Myofibers perpendicular to the B_0_ appear diamagnetic while those parallel to B_0_ are paramagnetic relative to the reference susceptibility [72]. In the healthy heart the contribution of collagen to magnetic susceptibility anisotropy is low, however its contribution does become significant (anti-parallel and ~ 50% magnitude of myofilament) in collagenised scars such as those found with ischemic cardiomyopathy [73] This makes STI a promising alternative technique, if the requirement for multiple B_0_ orientations can be overcome, to the most commonly used technique for mapping myofibre orientation, diffusion tensor imaging (DTI) [74]. While high-resolution cardiac DTI provides exclusively structural information (myofiber orientation), STI can combine structural and biochemical information including myocardial oxygenation and collagen content (myocardial scarring). Moreover, cardiac DTI suffers from spatial resolution limits (typically ~ 2.7 × 2.7 × 6 mm [74]), long scan times (e.g. 18 heartbeats per slice measurement in Nielles-Vallespin et al. [75]) and a low signal-to-noise ratio requiring multiple averages [75, 76]. On the other hand, STI is relatively novel, and less well validated compared to DTI, and STI reconstruction can suffer from low-frequency artefacts in the reconstructed tensor images. Several methods have been proposed to implement STI, including the incorporation of magnitude-derived relaxation and phase-derived susceptibility tensors [71], and the use of a gadolinium-based contrast agent. Gadolinium-based contrast-agent distributes in the extracellular space in normal myocardium causing rapid relaxation of water in this compartment without any change in the magnetic properties of the intracellular water. Thus, the use of gadolinium-based contrast-agent has the potential to minimise the signal from the extracellular space leaving the anisotropic intracellular compartment to dominate tissue susceptibility [77].

## QSM for atherosclerotic plaque assessment

**Fig. 4:**
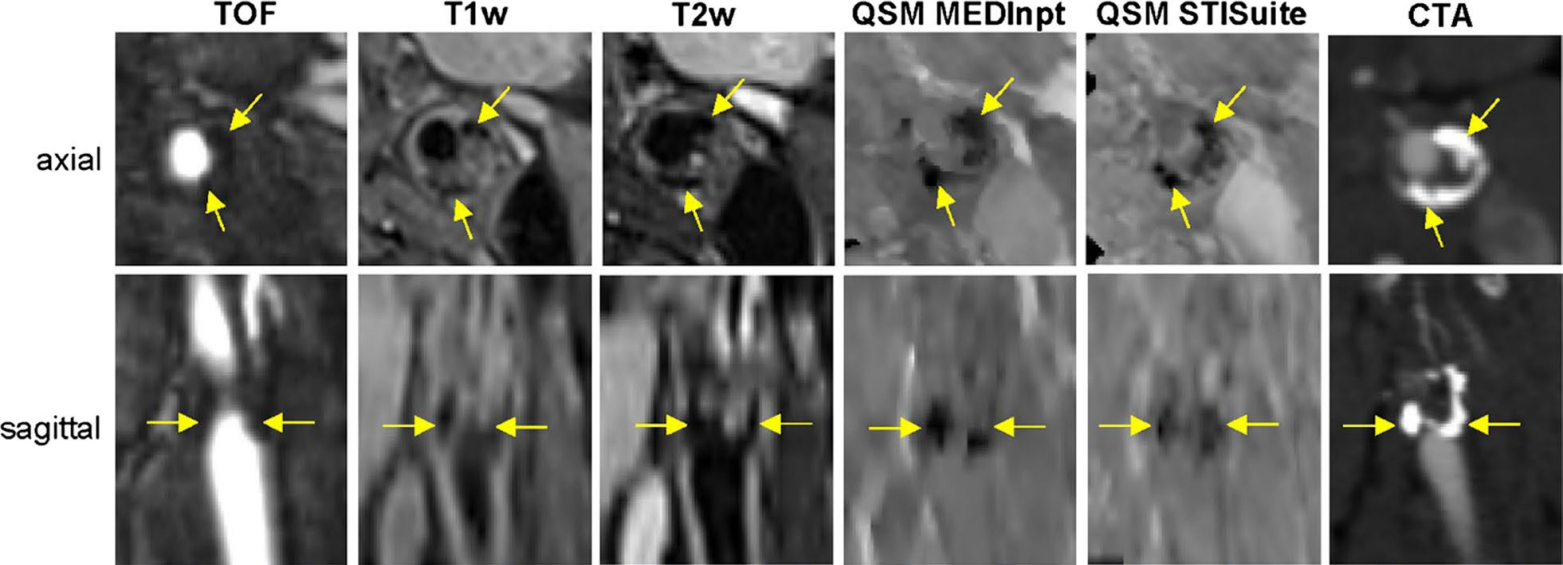
Quantitative susceptibility mapping (QSM) of a carotid plaque. Example of magnetic resonance imaging of a heavily calcified plaque almost fully occluding the left internal carotid artery at the level of carotid bifurcation with hypointense appearance on time-of-flight (TOF) and black-blood T_1_weighted (T_1_w) and T_2_-weighted (T_2_w) images as well as strongly negative susceptibility on QSM images computed with MEDInpt and the STISuite. The computed tomography angiography (CTA) image acquired 1.5 years before CMR at 0.6-mm resolution from the same patient is shown to help with plaque localization (Reprinted with permission from: Nguyen et al. [82])

Vulnerable atherosclerotic plaques are characterized by the presence of a lipid-rich necrotic core, intraplaque hemorrhage, a thin ruptured fibrous cap, and to a lesser degree calcification [78]. Currently, multi-contrast CMR is customary for carotid plaque characterization, with a focus on intra-plaque hemorrhage which is associated with rapid plaque progression and recurrent acute cerebrovascular events in patients with high-grade stenosis [79, 80]. Fresh intraplaque hemorrhage can be identified as a vessel wall region with hyperintense signal on T_1_-weighted images, while chronic intraplaque hemorrhage appears markedly hypointense on T_1_-weighted, T_2_- or T_2_* weighted, and time-of-flight images. The differentiation of chronic intraplaque hemorrhage and plaque calcification is challenging, given that both are associated with signal loss on multi-contrast CMR. In contrast, QSM can distinguish diamagnetic calcification from paramagnetic intraplaque hemorrhage [78, 81, 82], and strongly correlates with histology in patients undergoing carotid endarterectomy [83]. In this patient group calcified plaques had strongly negative susceptibility (≤ − 1 ppm), whereas intraplaque hemorrhage had positive susceptibility, ranging from ~ 0.5 ppm, in a recent haemorrhage, to 1.5–2 ppm in a chronic haemorrhage (Fig. 4).

Ultra-small superparamagnetic iron oxide (USPIO) contrast agents are increasingly used to measure macrophage infiltration. After intravenous injection, this compound enters plaque through its leaky endothelium and is internalised by macrophages. Comparison of pre- and post-USPIO iron-sensitive sequences, such as T_2_*weighted or R_2_*-maps, enables the quantification of the inflammatory component of atherosclerotic plaque. Recent studies indicate that QSM implemented with IDEAL water-fat separation can concurrently identify multiple high-risk plaque features (calcification, intraplaque haemorrhage, lipid-rich necrotic core and USPIO uptake), moreover, the addition of IDEAL improves QSM image quality reducing the streaking artefacts caused by chemical frequency shift. Thus, QSM processing could fit within a multi-contrast approach for quantification of atherosclerosis using a single 3D multi-echo GRE acquisition [84], eliminating the need for a time-consuming, and error-prone, multi-contrast CMR exam.

## Future challenges in cardiovascular QSM

Despite the recent breakthroughs in QSM, accurate quantification of magnetic susceptibility is still challenging. Existing methods need to be evaluated systematically under a variety of experimental conditions, and further work is needed to achieve high degrees of consistency. Blood flow and motion cause phase variations that can lead to erroneous magnetic field estimates. Chemical shift, proton exchange and partial volume effect can also influence frequency shift, introducing further errors. Currently, 3D multi-echo GRE acquisition, with a diaphragmatic navigator, is preferred to the breath-hold multi-slice 2D approach as this prevents slice misalignment and non-continuous k-space sampling. However, standard diaphragmatic navigators rely on a tracking factor (commonly set to 0.6) to correct for diaphragmatic and heart motions. Typical navigator gating efficiency (~ 25–60%) and the tracking factor is known to be subject-dependent and highly variable. Prospective respiratory gating techniques may provide higher gating efficiency (up to 100%) and improve motion correction using complex subject-specific models of the diaphragmatic/heart motion relationship [85–87] or image-based navigators which directly track heart motion [88, 89]. However, the respiration induces local magnetic susceptibility variations which may introduce errors in the QSM reconstruction if data are prospectively acquired at different respiratory phases.

Epicardial fat represents another challenge in cardiovascular QSM. However, Dixon-based fat-water separation methods, such as IDEAL, have been successfully adopted in abdomen or neck QSM [62, 84] and may offer advantages for cardiac QSM as well. However, these techniques rely upon prior knowledge of chemical spectrum which may influenced by a variety of factors such as lipid compartmentalization and fatty acids content. Recent techniques have been proposed to supersede this limitation, by correcting QSM for chemical shift map during reconstruction [63]. Another challenge to cardiovascular QSM is the high susceptibility at the interface of cardiac structures and lungs. However, several methods have been successfully applied for background phase removal in neck and abdominal QSM [62, 84] which could be integrated in the cardiovascular QSM pipeline, compensating for deficient B_0_ shimming [43]. Nonetheless, the numerical inconsistency of QSM in varying B_0_ shimming conditions still exists due to short T_2_* decay, and this issue is particularly relevant in higher magnetic fields (3T/7T), requiring higher-order shimming. Most current cardiovascular QSM literature is based on single-centre studies and there is currently insufficient data characterizing the technique’s accuracy, repeatability, and reproducibility (both within a given site/scanner and between sites/scanners). Different experimental setups may influence QSM, including the choice of the QSM reconstruction algorithm, imaging parameter selection, field strength, and shimming approaches/shim set performance. Therefore, the development of a commercially available QSM phantom together with the site-specific estimation of reference QSM values in healthy subjects/patients may be necessary for calibration and standardization purposes, as is currently the case in other areas such as myocardial T_1_ mapping [90]. Current reconstruction algorithms are often performed offline and retrospectively, due to computational cost. The design of fast reconstruction algorithms or the use of advanced computing hardware such as graphical processing units, increasingly available on commercial scanners, may represent a promising direction to reduce computational cost. Finally, machine learning techniques, based on convolutional neural networks are under active development for each stage of the QSM pipeline [91]. Examples include, PhaseNet 2.0 [92] for phase unwrapping, SHARQnet [93], for the separation of local and background fields, and QSMnet^+^ [94] for calculating magnetic susceptibility. These techniques may offer advantages over traditional algorithmic reconstruction techniques beyond reconstruction acceleration, including improved accuracy, robustness, and automation (e.g. automatic image segmentation), all of which have the potential to improve clinical utility and uptake.

## Conclusions

QSM is a promising method to expand the arsenal of measurable, reproducible and accurate CMR biomarkers in the heart, and therefore enable better understanding of the intricate pathophysiology processes underlying cardiovascular diseases. However, overcoming outstanding technical challenges and validation in large clinical studies are critical steps for a successful integration of cardiovascular QSM into clinical practice.

## Data Availability

Not applicable.

## Abbreviations

B_0_: Main magnetic field
CMR: Cardiovascular magnetic resonance
COSMOS: Calculation Of Susceptibility through a Multiple-Orientation Sampling
DTI: Diffusion tensor imaging
ECG: Electrocardiogram
GRE: Gradient echo
HEIDI: Homogeneity-Enabled Incremental Dipole Inversion
IMH: Intramyocardial haemorrhage
LBV: Laplacian Boundary Value
LV: Left ventricle/left ventricular
LVEF: Left ventricular ejection fraction
MEDI: Morphology Enabled Dipole Inversion
MRI: Magnetic resonance imaging
QSM: Quantitative susceptibility mapping
PDF: Projection onto Dipole Fields
ROI: Region of interest
SaO_2_: Oxygen saturation in the arterial blood
SHARP: Sophisticated Harmonic Artifact Reduction for Phase
STEMI: ST-segment elevation myocardial infarction
STI: Susceptibility tensor imaging
USPIO: Ultra-small superparamagnetic iron oxide
vSaO_2_: Mixed venous oxygen saturation
